# Comparison of acute single versus multiple osteoporotic vertebral compression fractures in radiographic characteristic and bone fragility

**DOI:** 10.1186/s13018-023-03874-7

**Published:** 2023-05-27

**Authors:** Feng Wang, Rui Sun, Shao-Dong Zhang, Xiao-Tao Wu

**Affiliations:** 1 Department of Spine Surgery, Zhongda Hospital, School of Medicine, Southeast University, 87# Dingjiaqiao Road, Nanjing, 210009 China; 2 Surgery Research Center, School of Medicine, Southeast University, 87# Dingjiaqiao Road, Nanjing, 210009 China

**Keywords:** Osteoporotic vertebral compression fracture, Multiple fracture, Fragility fracture, Osteoporosis, Vertebral fracture cascades

## Abstract

**Background:**

Osteoporotic vertebral compression fractures (OVCF) are common in aged population with bone fragility. This study aimed to identify the radiographic and bone fragility characteristic of acute single and multiple OVCF.

**Methods:**

OVCF patients hospitalized in a spine center between June 2016 and October 2020 were retrospectively studied. Demographics, comorbidity, bone mineral density, spine trauma, duration of pre-hospital back pain, anatomical location and distribution pattern of OVCF, extent of vertebral marrow edema, and degree of vertebral compression of patients with multi-segment vertebral fractures (MSVF) were summarized and compared to those with single segment vertebral fractures (SSVF).

**Results:**

A total of 1182 patients with 1530 acute fractured vertebrae were included. There were 944 SSVF (79.9%) and 238 MSVF (20.1%) simultaneously involving two (MSVF-2) or three and more vertebra (MSVF-3/m). The Female-Male ratio was 4.4 and differed not significantly between SSVF and MSVF. Females in SSVF were younger than males while MSVF-2 tended to occur in older females. L1, T12, and L2 were the three most frequently fractured vertebra and MSVF involved more vertebra in thoracic and lumbar spine. 31.1% in MSVF-2 and 83.1% in MSVF-3/m had at least two vertebral fractures in adjacent. The fractured thoracolumbar vertebra in MSVF was less compressed than that in SSVF. Apparent spine trauma was reported by 61.4% of SSVF, 44.1% of MSVF-2, and 36.3% of MSVF-3/m, while early hospitalization with pre-hospital back pain ≤ 1 week was 58.9% in SSVF, 45.3% in MSVF-2, and 25.9% in MSVF-3/m. Only females aged 70–80 years old in MSVF-3/m showed lower baseline bone mineral density than in MSVF-2 and SSVF. MSVF were not associated with increased comorbidity of hypertension, diabetes, coronary heart disease, cerebral infarction, and chronic pulmonary disease.

**Conclusions:**

20% of acute OVCF can involve multiple vertebra without significant spine trauma or lower baseline bone mineral density. Multiple OVCF tend to occur in adjacent vertebra with less thoracolumbar vertebral compression but longer duration of pre-hospital back pain.

## Background

Osteoporotic vertebral compression fractures (OVCF) are common in aged population with bone fragility, causing back pain and deteriorating the quality of life worldwide [1, 2]. Although vertebroplasty and kyphoplasty have proved efficient in augmenting vertebrae and reducing pain [2], subsequent fractures are potentially increased in adjacent segments [3–6], urging a need to better understand the pathogenesis of OVCF. To date, in addition to osteoporotic bone fragility, multiple factors including geriatric comorbidities, such as coronary heart disease and chronic obstructive pulmonary disease, as well as life styles, such as chronic smoking and alcohol consumption, all have been shown to increase the risk of new vertebral fractures [6–8].

While vertebral fracture cascade will accumulatively result in compressed and then cemented vertebra at multiple segments, it is noticeable acute OVCF can simultaneously involve multiple vertebra prior to the first time vertebroplasty or kyphoplasty [9–11]. In contrast to the notion that cement augmentation potentiates new adjacent OVCF [3, 4, 6], some argued that vertebral fracture cascade might be attributed mainly to, if not all, the natural history of aging and degenerating spine [5]. Based on this scenario, vertebral fracture cascade might start from a certain segment of osteoporotic spine, usually the thoracolumbar vertebrae [12], then extend cranially or caudally to adjacent and remote segments, driven by multiple factors including aging [3, 6, 7], osteoporosis [3, 7, 8], spine trauma [9], comorbidity [8], life style [6, 7], as well as stress change in the segments adjacent to cement augmentation [13]. Therefore, as for those undergo acute multi-segment OVCF, there might be aggravation of certain risk factors that potentially accelerates and prolongs vertebral fracture cascade.

In this study, the demographics, comorbidity, spine trauma, bone mineral density (BMD), duration of pre-hospital back pain, anatomical location and distribution pattern of OVCF, extent of vertebral marrow edema, and degree of vertebral compression of patients with acute single and multiple OVCF were, respectively, analyzed and compared. By identifying the risk factors of OVCF simultaneously involving multiple vertebra, our study may help to better understand the development and mechanism of vertebral fracture cascade.

## Methods

### Study population

This study was approved by Ethic Committee for Clinical Research of Zhongda hospital affiliated to Southeast University (No. 2022ZDSYLL016-P01). Medical records and Magnetic Resonance (MR) imaging of patients received vertebroplasty or kyphoplasty from June 2016 to October 2020 in the spine center of Zhongda hospital were retrospectively studied. The inclusion criteria included: (1) aged ≥ 45 years old; (2) primary diagnosis of acute OVCF based on symptom of back pain and signal of vertebral marrow edema on MR imaging of thoracic and lumbar spine; (3) full medical records detailing demographics, comorbidity, type of spine trauma, and duration of back pain before hospitalization. Exclusion criteria included: (1) pathologic diagnosis of infection, hemangioma, multiple myeloma, metastatic tumors, and other pathological vertebral fractures; (2) previous vertebroplasty, kyphoplasty or spine fixation surgery; (3) incomplete medical records or MR imaging of thoracic and lumbar spine.

### Grouping and data collection

**Fig. 1:**
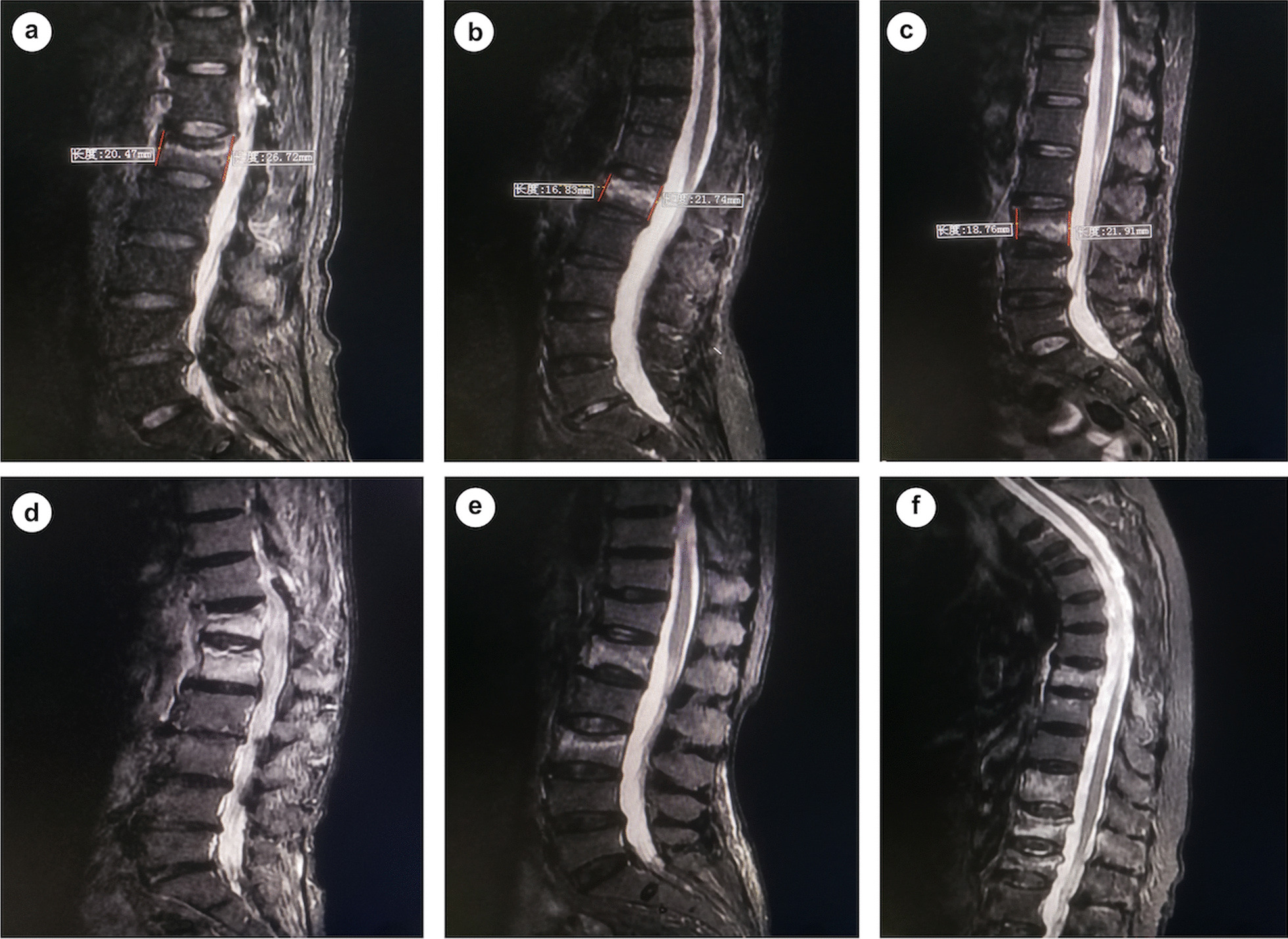
Radiographic characteristics of single and multiple osteoporotic vertebral compression fractures. The sagittal T2-weighted fat suppression MR imaging of single (**a**–**c**) and multiple (**d**–**f**) vertebral compression fractures. **a** A 78-year-old male complaining back pain for 5 days after fell on ground showed vertebral bone marrow edema in the cranial half of L1 vertebrae. The anterior and posterior height (red line) of fractured vertebrae was measured to quantify the degree of vertebral compression. **b** A 79-year-old female without spine trauma complained back pain for 1 week and showed diffused type of vertebral bone marrow edema in L1. **c** A 66-year-old female complained back pain for 4 days after heavy lift injury showed vertebral bone marrow edema in the caudal half of L3. **d** Two acute vertebral compression fractures in adjacent (T12 and L1) were detected in a 81-year-old male complaining back pain for 1 month without spine trauma. **e** Two acute vertebral compression fractures (L1 and L3) intermittent with one intact vertebrae (L2) were detected in a 74-year-old female complaining back pain for 1 month without spine trauma. **f** Four acute vertebral compression fractures (T8, T11, T12, and L1) with three in adjacent were detected in a 81-year-old female complaining back pain for 2 weeks without spine trauma

The study population was grouped according to the number of fractured vertebra: group of single segment vertebral fracture (SSVF) and group of multi-segment vertebral fractures (MSVF) involving two (MSVF-2) or three and more (MSVF-3/m) vertebra. Demographics (age, gender) and comorbidities (hypertension, diabetes mellitus, coronary heart disease, cerebral infarction, and chronic obstructive pulmonary disease) were collected from medical records. Segment of vertebral fracture was identified on MR imaging and in case of lumbosacral transitional vertebra reconfirmed by two radiologists. Based on the anatomical location of fracture, OVCF were divided into thoracic (T1–T9), thoracolumbar (T10–L2), and lumbar (L3–L5) group. Degree of vertebral compression was quantified by averaging the ratio of anterior and posterior height of the fractured vertebrae, which were measured on the sagittal MR imaging taken at mid-body and medial wall of two pedicles (Fig. 1). Extent of vertebral marrow edema was evaluated on the sagittal T2-weighted fat suppression MR imaging. Based on the location of edema signal within vertebrae, three types of vertebral marrow edema were defined: diffused type: edema detected both in the cranial and caudal half of vertebrae; cranial type: edema restrained in the cranial half of vertebrae; caudal type: edema restrained in the caudal half of vertebrae (Fig. 1). Spine trauma was divided into group of apparent trauma: fall on ground or crush injury to the spine; uncertain trauma: heavy lift injury, lumbar sprain, strenuous cough; no evident trauma. Duration of pre-hospital back pain was collected from patient’s chief complain and grouped into ≤ 1 week, 1–2 weeks (including the 2 weeks), 2–4 weeks, 1–3 months, and > 3 months. Bone mineral density (BMD) was quantified by the T-score values calculated from dual-energy X-ray absorptiometry (DXA) of lumbar spine and hip joint.

### Statistical analysis

Prism software (ver.9.1.2; Graphpad, San Diego, CA, USA) was utilized to perform statistical analysis. Descriptive statistics with Pearson *χ*^2^ were performed to compare the frequencies and percentages of categorical variables between the group of SSVF, MSVF-2, and MSVF-3/m. Continuous quantitative data were presented as means ± standard deviations. Differences between the group of SSVF, MSVF-2, and MSVF-3/m were analyzed by one-way ANOVA followed by Tukey’s multiple comparisons test. Statistical significance was defined as *P* value < 0.05.

## Results

### MSVF-2 tended to occur in older females

A total of 1490 vertebroplasty or kyphoplasty surgeries were performed in the spine center of Zhongda hospital from June 2016 to October 2020. A total of 1182 patients with 1530 acute fractured vertebra were included in this study. There were 944 SSVF, 161 MSVF-2, and 77 MSVF-3/m (Table 1).

**Table 1:** Clinical characteristics of single and multi-segment vertebral fractures

Clinical variables	SSVF	MSVF-2	MSVF-3/m	*P* value
	(*N* = 944)	(*N* = 161)	(*N* = 77)	
	*N* (%)	*N* (%)	*N* (%)	
Gender				0.996
Female	769 (81.46)	131 (81.37)	63 (81.82)	
Male	175 (18.54)	30 (18.63)	14 (18.18)	
Age for patients (in years)				0.083
< 60	113 (11.97)	17 (10.56)	9 (11.76)	
60–70	305 (32.31)	53 (32.92)	24 (31.32)	
70–80	313 (33.16)	38 (23.60)	28 (36.36)	
> 80	213 (22.56)	53 (32.92)	16 (20.78)	
Age for females (in years)				0.018
< 60	95 (12.35)	11 (8.40)	4 (6.35)	
60–70	263 (34.20)	44 (33.59)	24 (38.10)	
70–80	260 (33.81)	34 (25.95)	25 (39.68)	
> 80	151 (19.64)	42 (32.06)	10 (15.87)	
Age for males (in years)				0.025
< 60	18 (10.29)	6 (20.00)	5 (35.71)	
60–70	42 (24.00)	9 (30.00)	0 (0.00)	
70–80	53 (30.29)	4 (13.33)	3 (21.43)	
> 80	62 (35.43)	11 (36.67)	6 (42.86)	
Spine trauma				< 0.0001
Apparent trauma	580 (61.44)	71 (44.10)	28 (36.36)	
Uncertain trauma	112 (11.86)	12 (7.45)	11 (14.29)	
No evident trauma	252 (26.69)	78 (48.45)	38 (49.35)	
Duration of pre-hospital back pain				< 0.0001
≤ 1 week	556 (58.90)	73 (45.34)	20 (25.97)	
1–2 week	157 (16.63)	20 (12.42)	15 (19.48)	
2–4 week	134 (14.19)	37 (22.98)	12 (15.58)	
1–3 month	54 (5.72)	20 (12.42)	19 (24.68)	
> 3 month	43 (4.56)	11 (6.83)	11 (14.29)	
Type of comorbidity				
Hypertension	458 (48.52)	70 (43.48)	31 (40.26)	0.219
Diabetes	160 (16.95)	17 (10.56)	14 (18.18)	0.111
Coronary heart disease	117 (12.39)	17 (10.56)	8 (10.39)	0.725
Cerebral infarction	204 (21.61)	31 (19.25)	13 (16.88)	0.523
Chronic pulmonary disease	34 (3.60)	10 (6.21)	4 (5.19)	0.262
Number of comorbidity				0.443
0	359 (38.03)	69 (42.86)	37 (48.05)	
1	306 (32.42)	51 (31.68)	18 (23.38)	
2	187 (19.81)	30 (18.63)	14 (18.18)	
3	76 (8.05)	10 (6.21)	8 (10.39)	
4	16 (1.69)	1 (0.62)	0 (0.00)	

SSVF: Single segment vertebral fracture; MSVF-2: multi-segment vertebral fractures involving two vertebra; MSVF-3/m: multi-segment vertebral fractures involving three or more vertebra

The 1182 cases of OVCF included 963 females and 219 males. Female-Male ratio was 4.4 and differed not significantly between SSVF, MSVF-2, and MSVF-3/m (Table 1). The 1182 patients were averaged to 72.19 years old, and females (71.77 ± 9.34 years old) were on average 2.26 years younger than females (74.03 ± 10.85 years old). Females in SSVF (71.52 ± 9.37 years old) were 3.11 years younger than males (74.63 ± 10.29 years old) (*p* < 0.001). Females in MSVF-2 (73.37 ± 9.57 years old) were comparatively older than that in SSVF (*P* < 0.0001). The age ratio of patients was not significantly different between SSVF, MSVF-2, and MSVF-3/m. In MSVF the percentage of females < 60 years old decreased while that of males < 60 and > 80 years old increased (Table 1).

### MSVF involved more vertebra beyond thoracolumbar segment

**Fig. 2:**
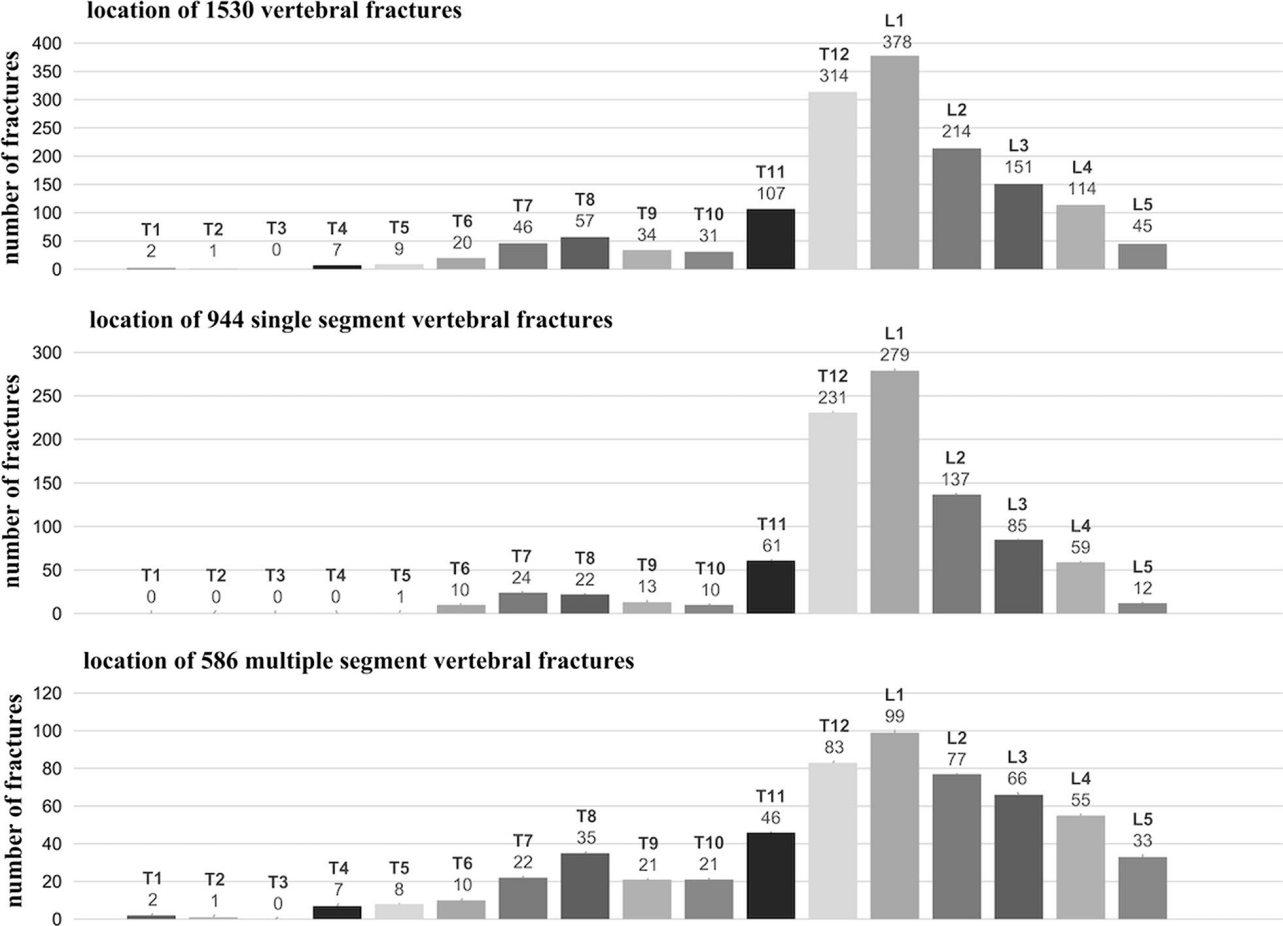
Distribution of single and multiple osteoporotic vertebral compression fractures. The 1530 fractured vertebrae from 1182 patients were unevenly located into thoracic (T1–T9), thoracolumbar (T10–L2), and lumbar (L3–L5) spine. L1, T12, and L2 were the three most frequently fractured vertebra in both single and multiple osteoporotic vertebral compression fractures. Multiple osteoporotic vertebral compression fractures involved more vertebra in lumbar and thoracic spine

The 1530 fractured vertebrae were unevenly located into thoracic (T1-T9), thoracolumbar (T10-L2), and lumbar (L3-L5) spine (Fig. 2). In both SSVF and MSVF, OVCF most frequently occurred in the thoracolumbar segment, with L1 having the highest incidence of vertebral fractures, followed by T12 and L2. T7, T8 and T9 were the three most frequently fractured vertebra in the thoracic spine. The incidence of OVCF decreased gradually from L3 to L5 in the lower lumbar spine (Fig. 2).

In MSVF, the ratio of vertebral fractures in thoracic and lumbar spine increased while that in thoracolumbar segment decreased (Table 2). The distribution pattern with at least two OVCF in adjacent was detected in 127 cases of MSVF (53.3%). MSVF-3/m had significantly higher ratio of vertebral fractures in adjacent than MSVF-2 (Table 3).

**Table 2:** Anatomical distribution of vertebral fractures and type of vertebral bone marrow edema

	SSVF	MSVF-2	MSVF-3/m	*P* value
	(*N* = 944)	(*N* = 322)	(*N* = 264)	
	*N* (%)	*N* (%)	*N* (%)	
Spine segment				< 0.0001
Thoracic	70 (7.42)	53 (16.46)	53 (20.08)	
Thoracolumbar	718 (76.06)	183 (56.83)	143 (54.17)	
Lumbar	156 (16.53)	86 (26.71)	68 (25.76)	
Vertebral marrow edema				0.271
Cranial type	158 (16.74)	49 (15.22)	48 (18.18)	
Diffused type	769 (81.46)	267 (82.92)	206 (78.03)	
Caudal type	17 (1.80)	6 (1.86)	10 (3.79)	

SSVF: single segment vertebral fracture; MSVF-2: multi-segment vertebral fractures involving two vertebra; MSVF-3/m: multi-segment vertebral fractures involving three or more vertebra

**Table 3:** Distribution pattern of multi-segment vertebral fractures

Distribution pattern	MSVF-2	MSVF-3/m	*P* value
	(*N* = 161)	(*N* = 77)	
	*N* (%)	*N* (%)	
Two OVCF in adjacent	63 (39.13)	64 (83.12)	< 0.0001
Intermittent with one intact vertebrae	40 (24.84)	7 (9.09)	
Intermittent with two or more intact vertebrae	58 (36.02)	6 (7.79)	

MSVF-2: multi-segment vertebral fractures involving two vertebra; MSVF-3/m: multi-segment vertebral fractures involving three or more vertebra

### Thoracolumbar vertebra in MSVF was less compressed than in SSVF

**Fig. 3:**
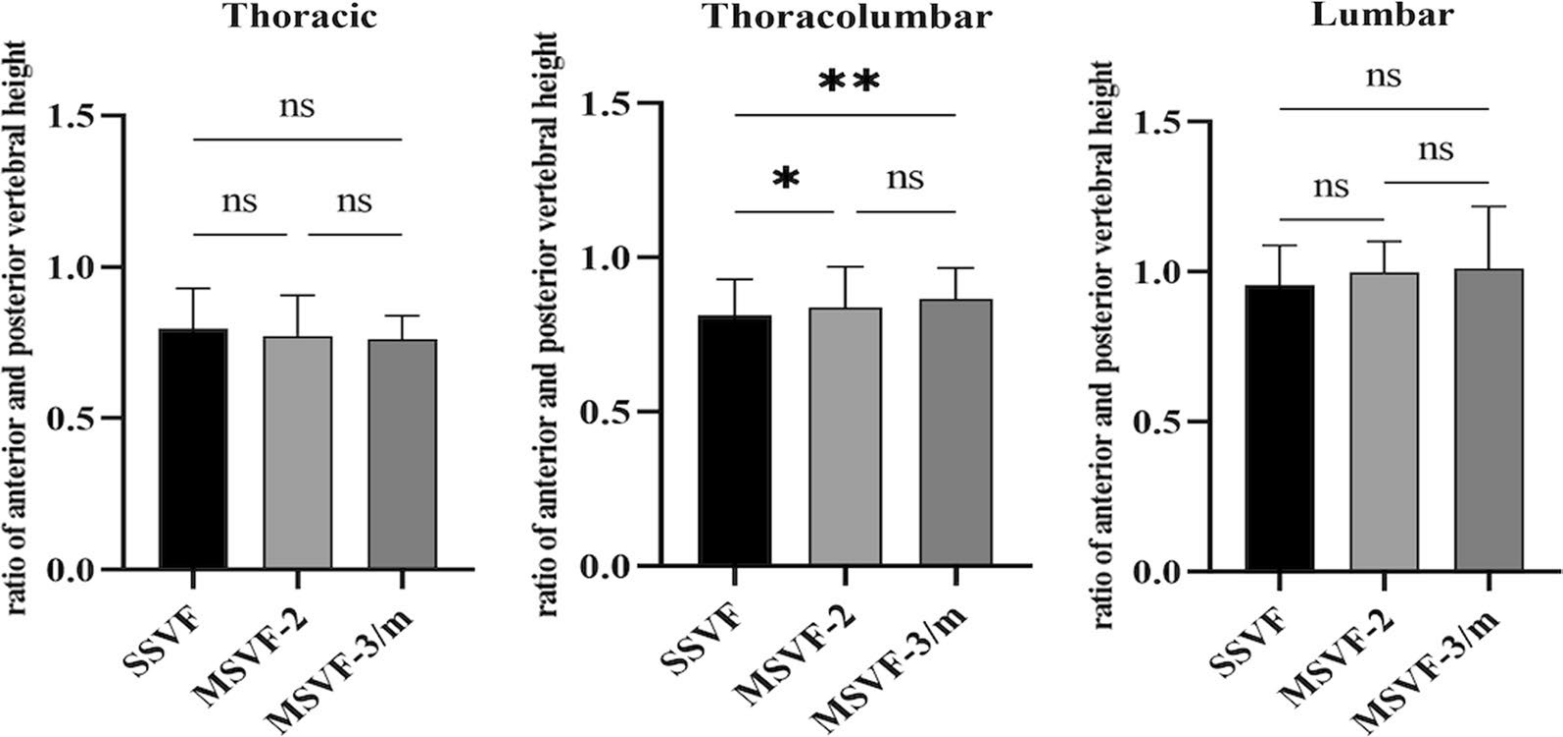
Degree of vertebral compression in single and multiple osteoporotic vertebral compression fractures. The ratio of anterior and posterior height of fractured thoracic, thoracolumbar, and lumbar vertebra. Thoracolumbar vertebra in MSVF-2 and MSVF-3/m showed significantly higher ratio of anterior and posterior vertebral height than in SSVF. SSVF: single segment vertebral fracture; MSVF-2: multi-segment vertebral fractures involving two vertebra; MSVF-3/m: multi-segment vertebral fractures involving three or more vertebra. ns: not significantly different; *p < 0.05; **p < 0.01

Of the 1530 fractured vertebrae, 1242 (81.2%) showed diffused type of vertebral marrow edema, 255 (16.6%) cranial type, and 33 (2.1%) caudal type. The ratio of edema type was not significantly different between SSVF, MSVF-2, and MSVF-3/m (Table 2). Due to the trapezoidal shape of lower lumbar vertebra (especially L5), degree of vertebra compression was evaluated, respectively, in thoracic, thoracolumbar, and lumbar spine. The fractured thoracolumbar vertebra in MSVF-2 and MSVF-3/m maintained higher ratio of anterior and posterior vertebral height than in SSVF. The thoracic and lumbar spine showed no significant difference in the degree of vertebral compression between SSVF and MSVF (Fig. 3).

### MSVF were not associated with severer spine trauma and lower baseline BMD

Of the 1182 cases of OVCF, 679 (57.4%) reported apparent trauma such as fall on ground and crush injury to the spine, 135 (11.4%) experienced uncertain trauma including heavy lift injury, lumbar sprain, and strenuous cough, and 368 (31.1%) denied evident trauma to the spine. MSVF-2 and MSVF-3/m reported lower ratio of apparent trauma but higher ratio of no evidence trauma than SSVF (Table 1).

**Fig. 4:**
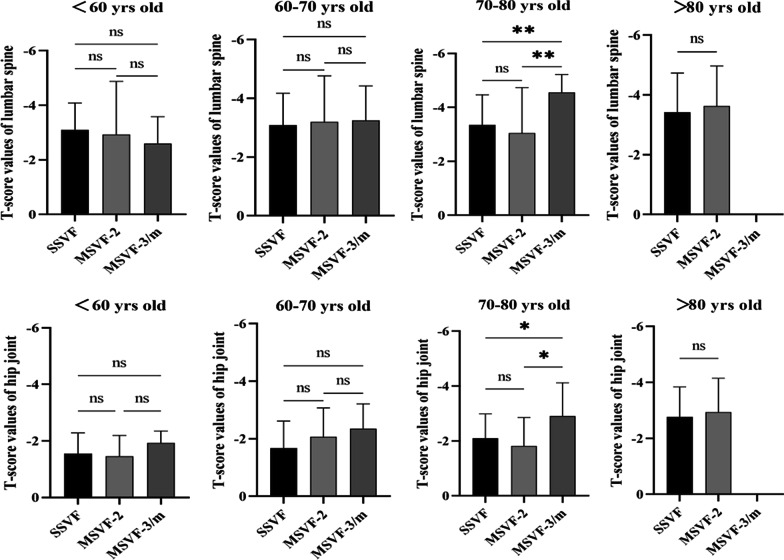
Bone mineral density of females with single and multiple osteoporotic vertebral compression fractures. The T-score values from dual-energy X-ray absorptiometry of lumbar spine and hip joint in 387 females with osteoporotic vertebral compression fractures. In the age group of 70–80 years old, MSVF-3/m showed significantly lower T-score values than MSVF-2 and SSVF. SSVF: single segment vertebral fracture; MSVF-2: multi-segment vertebral fractures involving two vertebra; MSVF-3/m: multi-segment vertebral fractures involving three or more vertebra. ns: not significantly different; *p < 0.05; **p < 0.01

BMD was available from 309 females in SSVF and 78 females in MSVF. The T-score values of lumbar spine (− 3.09 ± 1.45) and hip joint (− 1.91 ± 1.10) in SSVF were not significantly different from that (− 3.31 ± 2.29, − 2.23 ± 1.13) in MSVF (*P* > 0.05). Only the females aged 70–80 years old in MSVF-3/m showed lower T-score value of lumbar spine and hip joint than in MSVF-2 and SSVF. The age group of < 70 and > 80 years old showed no significant difference in the baseline BMD between SSVF and MSVF (Fig. 4).

### MSVF reported longer duration of pre-hospital back pain

The duration of pre-hospital back pain varied significantly from 1 day to more than 6 months. Of the 1182 cases of OVCF 649 (54.9%) were hospitalized early within 1 week since the onset of back pain, and 158 (13.3%) complained back pain for > 1 month before hospitalization. MSVF-2 and MSVF-3/m had lower ratio of early hospitalization within 1 week but higher ratio of pre-hospital back pain for > 1 month than SSVF (Table 1).

### MSVF were not associated with more geriatric comorbidities

Of the 1182 cases of OVCF, 559 reported comorbidity of hypertension, 191 of diabetes mellitus, 142 of coronary heart disease, 248 of cerebral infarction, and 48 of chronic obstructive pulmonary disease, averaging to a rate of one comorbidity per patient. A total of 465 patients (39.3%) reported none of the five geriatric comorbidities and 375 (31.7%) had one of them. MSVF-2 and MSVF-3/m had similar type and number of comorbidity as that in SSVF (Table 1).

## Discussion

In this retrospective study, we found that nearly 20% (238/1182) of acute OVCF diagnosed by MR imaging simultaneously involved two or more vertebra. The incidence of multiple OVCF was quite near to the incidence of 1/5 of new fractures post-vertebroplasty [4, 14, 15]. Moreover, we for the first time showed that simultaneous two OVCF (67.6%) was the most common form of MSVF, and more than half (53.3%) of MSVF had at least two OVCF in adjacent. The propensity of adjacent new fractures was also reported in the vertebral fracture cascade after cement augmentation, and the risk of new OVCF would increase 2.5 fold once the number of pre-existing fractured vertebra increased by one level [16, 17]. Probably due to the tendency of paired fractures in adjacent, MSVF demonstrated the same distribution predominance as SSVF in the thoracolumbar segment, but involved more vertebra in the nearby lumbar and thoracic spine. Therefore, based on the similarities in occurrence rate and distribution pattern between MSVF and new OVCF post-vertebroplasty [4, 14–17], we presume there might be two models of vertebral fracture cascade. One might start from a thoracolumbar vertebral fracture and extend gradually beyond to the nearby lumbar and thoracic spine, and the other one might occur much promptly as the form of MSVF simultaneously involving two or more vertebra in adjacent.

While multiple risk factors have been revealed to be associated with post-vertebroplasty re-fracture [3–8, 11, 14–17], it remains elusive what potentiates acute MSVF. Here, we found that females had 3.4-fold higher risk of vertebral fractures and experienced SSVF 3.1 years earlier than males. However, females rather than males in MSVF-2 were comparatively older than in SSVF, suggesting a risk factor of increasing age for acute MSVF in females. This is in line with previous report that older females with lower baseline BMD were at higher risk of repeated and multiple vertebral fractures [9, 16]. As multiple vertebral fractures were reported to be often caused by high-energy trauma [9], it is possible acute MSVF might experience higher incidence of spine trauma than SSVF. However, just on the contrary, we found that fewer MSVF reported apparent trauma such as fall on ground or crush injury to the spine, but more often denied evident spine trauma prior to the onset of back pain. Evidence accumulates that deteriorating osteoporosis promotes new fractures post-vertebroplasty [3, 8, 9, 16–19], and it is possible acute MSVF might have lower baseline BMD than SSVF. Here, BMD index was collected from 387 females (32.7% of study population), and only in the age group of 70–80 years old, MSVF-3/m showed lower T-score values than MSVF-2 and SSVF. Besides, comparison of comorbidities also identified no significant difference in the comorbidity type and number between MSVF and SSVF. Our study demonstrated that MSVF tended to occur in older females without significant spine trauma, lower baseline BMD, or multiple geriatric comorbidities. Vertebral fracture cascade in the form of acute MSVF might be driven by gender- and age-specific synergistic interactions of diverse risk factors.

OVCF are most often hospitalized with a complain of acute or chronic back pain [1, 2]. In this study of 1182 OVCF, the duration of pre-hospital back pain varied significantly from 1 day to longer than 6 months. While more than half of SSVF (58.9%) were hospitalized early with back pain ≤ 1 week, both MSVF-2 (19.2%) and MSVF-3/m (38.9%) reported higher ratio of pre-hospital back pain > 1 month and delayed hospitalization. One reason for the delay might be the fact that MSVF tended to occur in older females without significant spine trauma. In much aged patients with multiple comorbidities, prolonged observation of the back pain or conservative therapy would probably lead to delayed diagnosis and vertebroplasty in MSVF. As delayed vertebroplasty was reported to increase the risk of less maintained vertebral height and re-fracture [20, 21], it is also possible new vertebral fracture might occur during the observation or conservative therapy of back pain initiated by previous OVCF. In support of this hypothesis, although the ratio of vertebral bone marrow edema was not significantly different between SSVF and MSVF, MSVF-3/m caused more bone marrow edema restrained in the cranial or caudal half of vertebrae. Besides, the thoracolumbar vertebra in MSVF showed less compression and loss of anterior vertebral height than in SSVF. Collectively, our findings indicated a feature of inconspicuous but accelerated vertebral fracture cascade in the form of acute MSVF, highlighting a challenge of early diagnosis and vertebroplasty to alleviate back pain.

Our study was limited in its retrospective nature and relatively small number of included patients from a single spine center. The duration of pre-hospital back pain and type of spine trauma were based on patients’ chief complains after hospitalization, which unavoidably yielded lead time bias and recall bias [22]. Other risk factors of vertebral fracture cascade such as regional or global kyphosis [23], anti-osteoporosis medication [24–27], body mass index and life styles [6–8] were not evaluated to fully understand the mechanism of OVCF involving multiple vertebra. BMD was unavailable in 2/3 of the study population and in most of the males, which might underestimate the role of advanced osteoporosis in promoting MSVF. Recent evidence shows that biochemical marker of bone turnover is useful for evaluating bone fragility and therapy monitoring in postmenopausal osteoporosis [28, 29]. Further prospective studies with stratified participants and long-term follow-up are warranted for a better understanding of vertebral fracture cascade in the form of acute MSVF.

## Conclusions

20% of acute OVCF can involve multiple vertebra without significant spine trauma or lower baseline bone mineral density. Multiple OVCF tend to occur in adjacent vertebra with less thoracolumbar vertebral compression but longer duration of pre-hospital back pain.

## Data Availability

The datasets used and analyzed during the current study are available from the corresponding author on reasonable request.

## Abbreviations

OVCF: Osteoporotic vertebral compression fractures
SSVF: Single segment vertebral fracture
MSVF-2: Multi-segment vertebral fractures involving two vertebra
MSVF-3/m: Multi-segment vertebral fractures involving three or more vertebra
BMD: Bone mineral density
